# NdhM Subunit Is Required for the Stability and the Function of NAD(P)H Dehydrogenase Complexes Involved in CO_2_ Uptake in *Synechocystis* sp. Strain PCC 6803* [Fn FN2]

**DOI:** 10.1074/jbc.M115.698084

**Published:** 2015-12-24

**Authors:** Zhihui He, Min Xu, Yaozong Wu, Jing Lv, Pengcheng Fu, Hualing Mi

**Affiliations:** From the ‡ National Key Laboratory of Plant Molecular Genetics, Institute of Plant Physiology and Ecology, Shanghai Institutes for Biological Sciences, Chinese Academy of Science, 300 Fenglin Road, Shanghai 200032, China and; § Renewable Energy Research Center, China University of Petroleum Beijing, 18 Fuxue Road, Changping, Beijing 102249, China

**Keywords:** carbon fixation, cyanobacteria, electron transfer complex, photosynthesis, respiration

## Abstract

The cyanobacterial type I NAD(P)H dehydrogenase (NDH-1) complexes play a crucial role in a variety of bioenergetic reactions such as respiration, CO_2_ uptake, and cyclic electron transport around photosystem I. Two types of NDH-1 complexes, NDH-1MS and NDH-1MS′, are involved in the CO_2_ uptake system. However, the composition and function of the complexes still remain largely unknown. Here, we found that deletion of *ndhM* caused inactivation of NDH-1-dependent cyclic electron transport around photosystem I and abolishment of CO_2_ uptake, resulting in a lethal phenotype under air CO_2_ condition. The mutation of NdhM abolished the accumulation of the hydrophilic subunits of the NDH-1, such as NdhH, NdhI, NdhJ, and NdhK, in the thylakoid membrane, resulting in disassembly of NDH-1MS and NDH-1MS′ as well as NDH-1L. In contrast, the accumulation of the hydrophobic subunits was not affected in the absence of NdhM. In the cytoplasm, the NDH-1 subcomplex assembly intermediates including NdhH and NdhK were seriously affected in the Δ*ndhM* mutant but not in the NdhI-deleted mutant Δ*ndhI. In vitro* protein interaction analysis demonstrated that NdhM interacts with NdhK, NdhH, NdhI, and NdhJ but not with other hydrophilic subunits of the NDH-1 complex. These results suggest that NdhM localizes in the hydrophilic subcomplex of NDH-1 complexes as a core subunit and is essential for the function of NDH-1MS and NDH-1MS′ involved in CO_2_ uptake in *Synechocystis* sp. strain PCC 6803.

## Introduction

Thylakoid membranes of cyanobacteria contain a type I NAD(P)H dehydrogenase (NDH-1)^2^ complex homologous to complex I (NADH:ubiquinone oxidoreductase) from mitochondria and eubacteria (1, 2). The NDH-1 complexes in cyanobacteria are involved in respiration and cyclic electron transport (CET) around photosystem I (PSI) (3–7). In addition, cyanobacterial NDH-1 complexes function in inorganic carbon concentrating mechanisms (3, 8).

In cyanobacteria, 11 genes, *ndhA–ndhK*, encode proteins homologous to subunits of *Escherichia coli* complex I. However, no homologues to the *E. coli* genes encoding subunits NuoE, NuoF, and NuoG have been found in the cyanobacterial genomes, which contain the NADH-binding site, FMN cofactor, and Fe-S clusters essential for the bioenergetic function of complex I (9, 10). Four additional subunits (NdhL–NdhO) have been identified in *Synechocystis* sp. PCC 6803 (*Synechocystis* 6803) by a functional proteomics approach (11, 12). Further electron microscopy investigations revealed that the NdhL–NdhO subunits are located together, constituting the oxygenic photosynthesis-specific domain in *Synechocystis* 6803 (13). However, it was found that the NdhO is a new subunit that destabilizes the NDH-1 complex and represses its activity (14). There are five *ndhD* and three *ndhF* genes in *Synechocystis* 6803 (CyanoBase, the genome database for cyanobacteria). Different NDH-1 complexes consist of different types of NdhD and NdhF subunits, which are involved in diverse physiological functions. Four types of cyanobacterial NDH-1 complexes have been defined by reverse genetics (15, 16) and functional proteomics (11, 12). The large size NDH-1 complex (NDH-1L) containing NdhD1/NdhF1 and NDH-1L′ complex containing NdhD2/NdhF1 are involved in respiration and NDH-1-dependent CET around PSI (8, 17). NDH-1L complex is the predominating complex in the thylakoid membrane, and its expression is stable under different growth conditions; however, the NDH-1L′ complex has never been detected on the protein level (18). Nowaczyk *et al.* (19) reported two novel small subunits, NdhP and NdhQ, which were included in the purified NDH-1L complex by Ni^2+^ affinity chromatography and size exclusion chromatography from *Thermosynechococcus elongatus*. Recently, it has been demonstrated that NdhP is involved in respiration and CET and is essential to stabilize the NDH-1L complex (20–22).

All of these NDH-1 complexes contain a medium size NDH-1 complex (NDH-1M). One type of NDH-1 complex, the NDH-1MS complex, inducible at limiting inorganic carbon conditions, has a high uptake affinity for CO_2_, and is easily dissociated into NDH-1M and a small size NDH-1 complex (NDH-1S) (8, 16, 23). The expression of *ndhF3-ndhD3-cupA-sll1735* operon was induced when the cells of both *Synechocystis* 6803 and *Synechococcus* sp. PCC 7002 were grown under low CO_2_ condition (3). Further research showed that the proteins encoded by *ndhF3-ndhD3-cupA-sll1735* formed NDH-1S complex in which CupA and a small protein, CupS, were identified as subunits of cyanobacteria NDH-1S by proteomics analysis (18, 24). Because the NdhB-defective mutant M55 could not survive under low CO_2_ condition even when NDH-1S is present, it has been suggested that the normal operation of CO_2_ uptake system requires both NDH-1M and NDH-1S (18). The NDH-1MS complex has been isolated from a *T. elongatus* strain in which the C terminus of NdhL had been tagged with His_6_. This complex is easily dissociated into NDH-1M and NDH-1S complexes (24). NDH-1MS has been characterized as a U-shaped structure by single particle electron microscopy analysis after purification from the thylakoid membranes of *T. elongatus* (25). CupA is responsible for the U-shape by binding at the tip of the membrane-bound arm of NDH-1MS in both *T. elongatus* and *Synechocystis* 6803 (26). As a homologous gene of *cupA*, *cupB* (*chyX*) is involved in the constitutive CO_2_ uptake system that encoded by *ndhD4*/*ndhF4*/*cupB* and forms a small complex, NDH-1S′ (16, 23). It has been found that CupB protein is located in thylakoid membrane but is absent in that of NdhD4-deleted mutant (27). Based on the fact that the purified 450-kDa complex contained both NdhH and CupB proteins, it has been suggested that the complex is NDH-1MS′ located in the thylakoid membranes. However, so far, the composition and the function of NDH-1MS and NDH-1MS′ still remain to be elucidated.

Despite extensive biochemical and genetic studies of the cyanobacterial NDH-1 complexes, the enzymatic activity of the NDH-1 complex remains elusive, and the electron donor for the cyanobacterial NDH-1 complexes is still unclear. An *in vitro* experiment indicated that the electron donation occurs from reduced ferredoxin to the plastoquinone via NDH (5). It has been reported that the newly identified NdhS subunits from *Arabidopsis thaliana* (also known as CRR31) and from *Synechocystis* 6803 contain a Src homology 3 domain-like fold, which serves as the ferredoxin docking site domain (28–30), and it was suggested that the chloroplast NDH complex could accept electrons from ferredoxin rather than NAD(P)H. Recently, we have found that the NDH-1L complex interacts with ferredoxin via the subunit NdhS in *T. elongatus* (31).

The NdhM subunit of NDH-1 was first detected by immunoprecipitation experiments using antibodies specific for NdhM in *Synechocystis* 6803 (32). A proteomics study confirmed that the NdhM subunit was one of the subunits of NDH-1 complex (11). In higher plants, knock-out of *ndhM* results in complete impairment of NDH activity and the entire collapse of subcomplex A of chloroplast NDH complex (33–35). However, the function of the cyanobacterial NdhM subunit and its localization in NDH-1 complexes are still unclear. Here, we demonstrate that deletion of *ndhM* gene disassembled the hydrophilic subcomplex of NDH-1L, Ndh-1MS, and NDH-1MS′, resulting in the inactivation, of CO_2_ uptake, CET, and respiration. Based on the results of *in vitro* protein-protein interaction and the assembly intermediates of NDH-1 subcomplex, we propose models for the localization of NdhM in the NDH-1 complexes required for respiration, CET around PSI, and CO_2_ uptake.

## Experimental Procedures

### 

#### 

##### Culture Conditions

The glucose-tolerant strain of wild-type (WT) *Synechocystis* 6803 and mutants Δ*ndhK* (36) and M55 (3) were cultured at 30 °C in BG-11 medium (37) buffered with Tris-HCl (5 mm, pH 8.0) and bubbled with 5% (v/v) CO_2_ in air. The solid medium used was BG-11 supplemented with 1.5% agar. Continuous illumination was provided by fluorescence lamps at 40 μmol of photons m^−2^ s^−1^.

##### Construction of ΔndhM and ΔndhI Mutants

**FIGURE 1.**
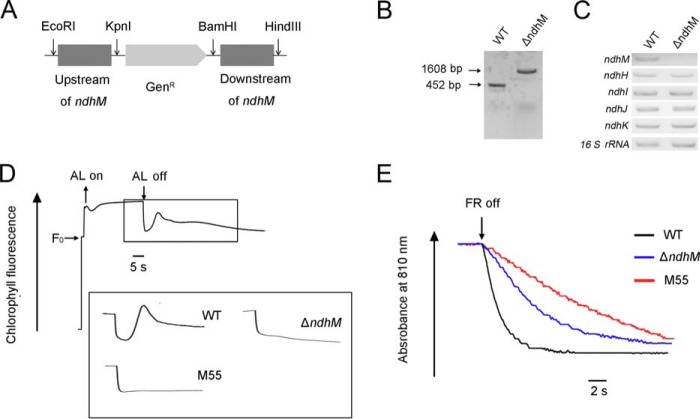
***ndhM* gene deletion and its effect on NDH-1 activity.** *A*, construction of plasmid to generate *ndhM* deletion mutant (Δ*ndhM*). A schematic representation of the Δ*ndhM* mutant locus is shown. A gentamicin resistance (*Gen^R^*) marker was used to replace the entire *ndhM* gene. *B*, PCR segregation analysis of the Δ*ndhM* mutant using the *ndhM*-F and *ndhM*-R primers (supplemental Table 1). *C*, transcript abundance of *ndhM*, *ndhH*, *ndhI*, *ndhJ*, and *ndhK* genes in the WT and Δ*ndhM* mutant. The abundance of the 16 S rRNA is shown as a control. *D*, monitoring of NDH-1 activity using chlorophyll fluorescence analysis. The *upper* curve shows a typical trace of chlorophyll fluorescence in WT *Synechocystis* 6803. Cells were exposed to actinic light (*AL*) (60 μmol of photons m^−2^ s^−1^) for 60 s, and after it was turned off, the transient increase in chlorophyll fluorescence level was ascribed to NDH activity. The *inset* shows magnified traces from the *boxed* area. *E*, kinetics of P700^+^ re-reduction in darkness after turning off far-red light (*FR*) in the presence of 10 μm 3-(3,4-dichlorophenyl)-1,1-dimethylurea. The chlorophyll *a* concentration was adjusted to 30 μg ml^−1^, and curves are normalized to the maximal signal.

The upstream and downstream regions of *slr1623* (*ndhM*) were amplified by PCR, creating appropriate restriction sites. A DNA fragment encoding a gentamicin resistance (Gen^R^) cassette was also amplified by PCR, creating KpnI and BamHI sites using specific oligonucleotide primers (supplemental Table 1). These three products were ligated into the multiple cloning site of pUC19 (Fig. 1*A*), which was used to transform the WT cells of *Synechocystis* 6803 as described by Williams and Szalay (38). The transformants were spread on agar plates containing BG-11 medium and gentamicin (10 μg ml^−1^) buffered at pH 8.0, the plates were incubated in 2% (v/v) CO_2_ in air, and continuous illumination was provided by fluorescence lamps at 40 μmol of photons m^−2^ s^−1^. The mutated *ndhM* in the transformants was segregated to homogeneity as determined by PCR amplification and immunoblotting. The same strategy was used for constructing the Δ*ndhI* mutant.

##### RNA Extraction and RT-PCR Analysis

Cells were harvested from 50 ml of cultures at 4 °C and treated with TRIzol reagent (Thermo Fisher Scientific) according to the provided protocol. RT-PCR was performed using the RT-PCR system (Promega) to generate products corresponding to *ndhM*, *ndhH*, *ndhI*, *ndhJ*, *ndhK*, and 16 S rRNA with 0.5 μg of DNase-treated total RNA as starting material. RT-PCR conditions were 95 °C for 5 min followed by cycles of 95 °C for 30 s, 58 °C for 20 s, and 72 °C for 1 min. The reactions were stopped after 16 cycles for 16 S rRNA and after 38 cycles for *ndhM*, *ndhH*, *ndhI*, *ndhJ*, and *ndhK.* The primers used are summarized in supplemental Table 1.

##### The Measurement of Chlorophyll Fluorescence and Redox Kinetics of P700

The transient increase in chlorophyll fluorescence after actinic light had been turned off was monitored by means of a PAM chlorophyll fluorometer (Walz, Effeltrich, Germany), emitter-detector-cuvette assembly (ED-101US), and an 101ED unit as described previously (5, 39). After dark acclimation for 30 min, samples were exposed to actinic red light (∼630 nm; 60 μmol of photons m^−2^ s^−1^) for 60 s, and the kinetics of photosystem II (PSII) chlorophyll fluorescence after switching off actinic illumination was recorded as a measure of NDH activity. Minimal fluorescence at open PSII centers in the dark-adapted state (initial fluorescence (*F*_0_)) was excited by a weak measuring light (600 nm). A saturating white light (1500 μmol of photons m^−2^ s^−1^) was applied to simultaneously determine the maximal fluorescence (*F_m_*) at closed PSII centers.

The redox kinetics of P700 was measured as described previously (5, 39). The re-reduction of P700^+^ in darkness was measured using the PAM chlorophyll fluorometer, ED-101US, and an ED-P700DW-II unit by monitoring absorbance changes at 830 nm and using 875 nm as a reference. Cells were kept in the dark for 2 min, and 10 μm 3-(3,4-dichlorophenyl)-1,1-dimethylurea was added to the cultures prior to measurement. P700 was oxidized by far-red light (>720 nm; 16 μmol of photons m^−2^ s^−1^ from a light-emitting diode lamp for 40 s), and the subsequent re-reduction of P700^+^ in the dark was monitored.

##### CO_2_ Uptake Measurement

The CO_2_ uptake rate was determined using a portable open flow gas exchange system (Li-6400, LI-COR Biosciences). The WT, Δ*ndhM*, and M55 strains were grown at 30 °C in BG-11 medium, then collected by centrifugation, and resuspended in fresh growth medium at a chlorophyll *a* concentration of 400 μg ml^−1^. 30 μl of the cell suspension was placed on the agar plate (2 × 2 cm) to measure the CO_2_ uptake rate. Air temperature of the leaf chamber was maintained at 30 °C, the photosynthetically active radiation was 60 μmol of photons m^−2^ s^−1^, and the flow rate of the air in the measuring chamber was 50 μmol s^−1^. The CO_2_ concentration was controlled at 400 μmol mol^−1^. Measurements were repeated three times, and the averages were recorded.

##### Isolation of Crude Thylakoid Membranes

Thylakoid membranes from *Synechocystis* 6803 were isolated as described by Gombos *et al.* (40) with some modifications as follows. Cell cultures (1 liter) were harvested, resuspended in 5 ml of disruption buffer (10 mm HEPES-NaOH, 5 mm sodium phosphate, pH 7.5, 10 mm MgCl_2_, 10 mm NaCl, 20% (v/v) glycerol), broken by shaking with glass beads (150–212 μm) using the Tissuelyser-48 system (Shanghi Jingxin), and then centrifuged at 5,000 × *g* for 5 min at 4 °C to remove glass beads and unbroken cells. The crude thylakoid membranes were obtained by centrifugation of the supernatant at 20,000 × *g* for 30 min at 4 °C. The thylakoid membranes were suspended in solubilization buffer (20 mm BisTris-HCl, pH 7.0, 10 mm MgCl_2_, 20% (v/v) glycerol) at a final chlorophyll concentration of 1 mg ml^−1^.

##### Isolation of the Membrane and Soluble Cell Fractions

Total membrane and soluble fractions of *Synechocystis* 6803 cells were isolated as described by Dai *et al.* (41) with slight modifications. Cell cultures were harvested, resuspended in disruption buffer, broken by shaking with glass beads (150–212 μm), and then centrifuged at 5,000 × *g* for 5 min at 4 °C to remove glass beads and unbroken cells. The total membrane and soluble fractions were separated by centrifugation at 100,000 × *g* for 30 min.

##### Electrophoresis and Immunoblotting

Blue native (BN)-PAGE of the thylakoid membranes from *Synechocystis* 6803 was performed as described previously (42) with slight modifications as follows. Membranes were washed with 330 mm sorbitol, 50 mm BisTris-HCl, pH 7.0, and solubilized in 25 mm BisTris-HCl, pH 7.0, 10 mm MgCl_2_, 20% (v/v) glycerol at a chlorophyll *a* concentration of 0.5 mg ml^−1^. After incubation on ice for 40 min with 2% *n*-dodecyl β-d-maltoside and centrifugation at 20,000 × *g* for another 15 min, the supernatants were supplemented with 110 volume of BN sample buffer (5% Serva Blue G, 100 mm BisTris-HCl, pH 7.0, 30% (w/v) sucrose, 500 mm ϵ-amino-*n*-caproic acid, 10 mm EDTA). Solubilized membranes were then applied to a 0.75-mm-thick 5–13% acrylamide gradient gel. Electrophoresis was performed at 4 °C by increasing the voltage gradually from 50 to 200 V during the 5-h run. The lanes of the BN gel were cut out and incubated in Laemmli SDS sample buffer containing 5% β-mercaptoethanol for 30 min. SDS-PAGE of the membrane protein was performed on a 12% polyacrylamide gel as described previously (43).

Clear native (CN)-PAGE of the soluble cell fractions from *Synechocystis* 6803 was performed as described previously (35) with slight modifications. A total of 50 μg of cytoplasmic proteins was mixed with ¼ volume of sample buffer (40 mm BisTris-HCl, pH 7.0, 0.008% Ponceau S, 200 mm ϵ-amino-*n*-caproic acid, 60% (v/v) glycerol). Cytoplasmic proteins were separated by 5–13% acrylamide gradient CN-PAGE in 0.75-mm-thick gels. Electrophoresis was performed at 4 °C by increasing the voltage gradually from 50 to 200 V during the 4-h run. The lanes of the CN gel were cut out and incubated in Laemmli SDS sample buffer containing 5% β-mercaptoethanol for 30 min. SDS-PAGE of the proteins was performed on a 12% polyacrylamide gel.

For immunoblotting, the proteins in the gel were electrotransferred to a polyvinylidene difluoride (PVDF) membrane (Immobilon-P, Millipore) and detected using protein-specific antibodies with the ECL assay kit (Thermo Scientific) according to the manufacturer's protocol. Antibody against the NdhM protein of *Synechocystis* 6803 was raised in our laboratory. Primer sequences used to amplify the *ndhM* gene are listed in supplemental Table 1. The PCR product was ligated into vector pET28a (Novagen). The plasmid was used to transform *E. coli* strain BL21(DE3) pLysS for expression. Polyclonal antibody was raised in a rabbit from purified recombinant protein. The antibodies against NdhH, NdhI, NdhJ, NdhK, NdhA, CupA, and CupB were previously raised in our laboratory.

##### Expression and Purification of Fusion Proteins

For testing the direct interaction of NdhM with Ndh subunits, the fragments containing *ndhH*, *ndhI*, *ndhJ*, *ndhK*, *ndhN*, *ndhO*, and *ndhS* genes were amplified by PCR and cloned into pET28a, respectively, to form His-tagged fusion protein constructs. The fragment containing *ndhM* was cloned into pGEX-4T-1 to form the GST-tagged fusion construct. Primer sequences used are listed in supplemental Table 1. These constructs were transformed into *E. coli* strain BL21(DE3) pLysS and induced by 1 mm isopropyl β-d-thiogalactoside for 16 h at 16 °C. These fusion proteins were purified using a nickel column (GE Healthcare) and glutathione-Sepharose 4B column (GE Healthcare), respectively, according to the manufacturer's instructions.

##### GST Pulldown Assay

GST-NdhM or GST- and His-tagged fusion proteins were incubated with 20 μl of glutathione-Sepharose 4B resin for 2 h at 4 °C in a buffer containing 20 mm Tris-HCl, pH 7.5, 100 mm NaCl, 0.1 mm EDTA, 0.2% (v/v) Triton, 10% (v/v) glycerol. The protein-bound resin was washed five times with a buffer containing 20 mm Tris-HCl, pH 7.5, 300 mm NaCl, 0.1 mm EDTA, 0.5% (v/v) Nonidet P-40. The washed proteins were directly eluted with Laemmli buffer at 95 °C for 5 min. The input and eluates were analyzed by immunoblotting and Coomassie staining.

##### Yeast Two-hybrid Assay

The yeast two-hybrid assay was performed according to the yeast protocols (Clontech). The *ndhM* gene was amplified by PCR with primers listed in supplemental Table 1 and cloned into pGBKT7 vector (Gal4 DNA binding domain; Clontech) to form the bait construct. The *ndhH*, *ndhI*, *ndhJ*, *ndhK*, *ndhL*, *ndhN*, *ndhO*, and *ndhS* genes were cloned into the pGADT7 vector (Gal4 activation domain; Clontech) to form the prey constructs. To test protein interactions, plasmids were cotransformed into *Saccharomyces cerevisiae* AH109 cells. Successfully transformed colonies were identified on yeast dropout medium lacking Trp and Leu; these colonies were resuspended in water and transferred to selective medium lacking Trp, Leu, His, and Ade. Yeast cells were incubated at 30 °C for 6 days. Empty vectors were cotransformed as negative controls.

## Results

### 

#### 

##### Deletion of ndhM Impairs NDH-1 Activity

To investigate the function of *slr1623* (*ndhM*) gene in NDH-1 complexes, we replaced the *ndhM* coding region by a gentamicin resistance (Gen^R^) cassette (Fig. 1*A*). PCR analysis of the *ndhM* locus confirmed the complete segregation of the Δ*ndhM* allele (Fig. 1*B*). Transcript analysis demonstrated the absence of *ndhM* gene product in the mutant, whereas the expression of the *ndhH*, *ndhI*, *ndhJ*, and *ndhK* genes was not affected (Fig. 1*C*). Immunoblotting analysis using the antibody specifically prepared against NdhM demonstrated the absence of the gene product in this mutant (Fig. 3*A*). The transient increase in chlorophyll fluorescence after illumination with actinic light, which is attributed to the NDH-dependent non-photochemical reduction of the plastoquinone pool in the dark, is used as an indicator of NDH-1 activity (5, 39). In Δ*ndhM*, the transient increase of chlorophyll fluorescence was completely arrested, similar to that observed in the M55 mutant (Fig. 1*D*). This result indicates that the NDH-1 activity was severely affected in Δ*ndhM*. A similar result was obtained by measuring the re-reduction of P700^+^ in darkness. P700 was oxidized by far-red light (>720 nm) for 40 s, and then the subsequent re-reduction of P700^+^ in the dark was monitored. The operation of NDH-1 complexes transfers electrons from the reduced plastoquinone pool and accelerates the re-reduction of P700^+^ (5). The re-reduction rate of P700^+^ was much slower in Δ*ndhM* compared with that in the WT strain; however, it was relatively faster than the M55 mutant (Fig. 1*E*). These results confirmed that the deletion of *ndhM* impairs the NDH-1 activity.

We also examined the variable fluorescence (*F_v_*) and *F*_0_ levels of WT and Δ*ndhM*. In Δ*ndhM*, both the *F_v_* and *F*_0_ levels were similar to that in WT (Table 1). This result indicates that the PSII activity was not affected in the Δ*ndhM* mutant.

**TABLE 1:** **Chlorophyll fluorescence parameters** The values shown represent the mean ± S.D.; the experiment is the average of three independent measurements.

Strain	*F*_0_	*F_m_*	*F_v_*	*F_v_*/*F_m_*
WT	0.26 ± 0.01	0.71 ± 0.01	0.45 ± 0.01	0.63 ± 0.01
Δ*ndhM*	0.27 ± 0.01	0.71 ± 0.01	0.44 ± 0.01	0.62 ± 0.01

##### Inactivation of Both Activities of CO_2_ Uptake and Respiration in ΔndhM

**FIGURE 2.**
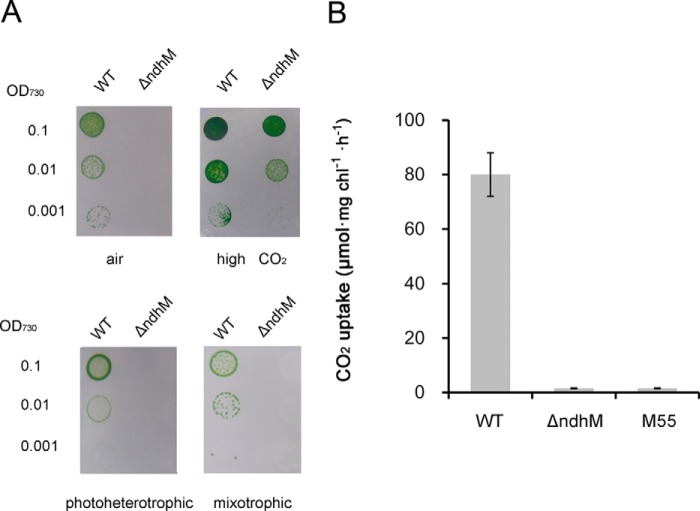
**Growth of WT and Δ*ndhM* strains on BG-11 agar plates with different carbon sources and the rates of CO_2_ uptake in WT and mutant strains.** *A*, growth of WT and Δ*ndhM* strains. The concentration of the cells was adjusted to OD_730_ = 0.1, 0.01, and 0.001. Three microliters of the cell suspensions was placed on the agar plate, and the cultures were grown for 7 days at 40 μmol of photons m^−2^ s^−1^. Conditions were as follows: *high CO_2_*, 2% (v/v) CO_2_ in air; *photoheterotrophic*, BG-11 medium + 5 mm glucose + 10 μm 3-(3,4-dichlorophenyl)-1,1-dimethylurea; *mixotrophic*, BG-11 medium + 5 mm glucose. *B*, the rate of CO_2_ uptake in WT, Δ*ndhM*, and M55 strains. The chlorophyll (*chl*) *a* concentration was adjusted to 400 μg ml^−1^. 30 μl of the cell suspensions was placed on the BG-11 agar plate. The CO_2_ uptake rate was measured at 30 °C. The CO_2_ concentration was controlled at 400 μmol mol^−1^. Light intensity was 60 μmol of photons m^−2^ s^−1^. The data shown are average values for three biological replicates, and the *error bars* show S.D.

In addition to CET around PSI, NDH-1 complexes are also involved in CO_2_ uptake and respiration (18). To examine CO_2_ uptake and respiration activities, WT and Δ*ndhM* mutant were grown on BG-11 plates in the presence of 2% CO_2_ in air or air as well as under photoheterotrophic and mixotrophic conditions. The growth rate of Δ*ndhM* was a bit slower than that of WT under autotrophic growth conditions supplied with 2% CO_2_ in air; however, it grew very slowly under the air condition. Meanwhile, Δ*ndhM* also grew very slowly in the presence of glucose under either the photoheterotrophic or mixotrophic condition (Fig. 2*A*). This result implies that NdhM is essential for respiration and CO_2_ uptake. To confirm that the CO_2_ uptake activity was affected in the Δ*ndhM* mutant, we measured the rates of CO_2_ uptake in WT, Δ*ndhM*, and M55 mutants using a portable open flow gas exchange system. Cell suspensions were placed on the BG-11 agar plate to measure the CO_2_ uptake rate. The rate of CO_2_ uptake was significantly lower in Δ*ndhM* than that in WT but similar to that in the M55 mutant (Fig. 2*B*). These results demonstrated that deletion of *ndhM* impaired both CO_2_ uptake and respiration activities.

##### Disassembly of NDH-L, NDH-1MS, and NDH-1MS′ in ΔndhM

**FIGURE 3.**
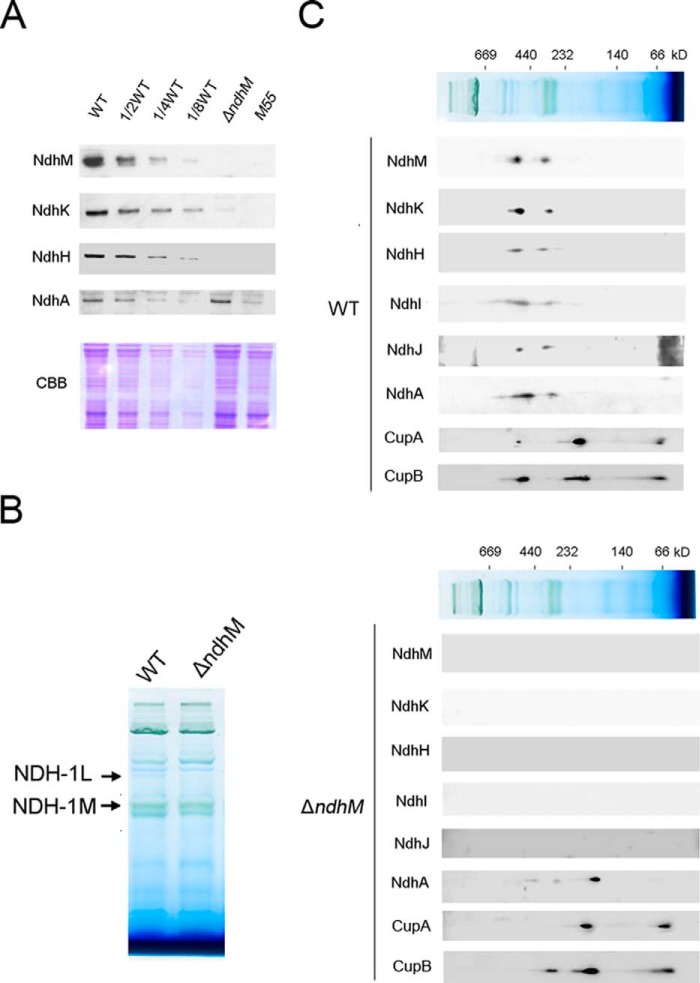
**Accumulation of Ndh subunits and their assembly into the NDH-1L and NDH-1 M complexes in the thylakoid membranes of WT, Δ*ndhM*, and M55 strains.** *A*, immunodetection of Ndh subunits in thylakoid membranes from WT (including indicated serial dilutions) and Δ*ndhM* and M55 mutants. Immunoblotting was performed using antibodies against Ndh subunits (NdhM, NdhK, NdhH, and NdhA). Lanes were loaded with thylakoid membrane proteins corresponding to 2 μg of chlorophyll *a*. In the *lower panel*, a replicated gel stained with Coomassie Brilliant Blue (*CBB*) was used as a loading control. *B*, profiles of BN-PAGE of thylakoid membranes from WT and Δ*ndhM* strains. Each lane was loaded with thylakoid membrane proteins corresponding to 6 μg of chlorophyll *a. Arrows* indicate the NDH-1L and NDH-1M complexes. *C*, thylakoid membrane proteins from WT and Δ*ndhM* strains were separated by BN-PAGE and further subjected to two-dimensional SDS-PAGE. The proteins were immunodetected with antibodies against the Ndh subunits (NdhM, NdhK, NdhH, NdhI, NdhJ, and NdhA), CupA, and CupB. The positions of molecular mass markers in the BN gel are indicated.

To investigate how NDH-1 activity is affected in the absence of NdhM, we compared the accumulation and assembly of the NDH-1 complex in the thylakoid membranes of the WT, Δ*ndhM*, and M55 strains by immunoblotting analysis. Results from Western blotting with antibodies against NdhM, NdhH, NdhK, and NdhA showed that inactivation of *ndhM* almost completely abolished accumulation of the NdhM, NdhH, and NdhK subunits in the hydrophilic part of NDH-1 complex; however, the accumulation of NdhA from the hydrophobic part in the membrane was not affected (Fig. 3*A*), whereas the accumulation of these Ndh subunits was nearly abolished in the M55 mutant.

Furthermore, to investigate how the NDH-1 complexes are affected in Δ*ndhM*, we separated the protein complexes from thylakoid membranes in Δ*ndhM* and WT by 5–13% gradient BN-PAGE. The result shows that the band corresponding to the NDH-1L complex disappeared in Δ*ndhM* compared with the WT (Fig. 3*B*). Further two-dimensional SDS-PAGE and immunoblotting analysis showed that the NDH-1L and NDH-1M complexes were both disassembled in the Δ*ndhM* mutant. All the hydrophilic subunits were absent in the NDH-1L and NDH-1M complexes. The NdhA subunit was present in a subcomplex with a molecular mass of ∼200 kDa consisting of the hydrophobic subunits only (Fig. 3*C*). Moreover, we also checked the low CO_2_-induced NDH-1MS and the constitutively expressed NDH-1MS′ complexes, which are essential for CO_2_ uptake activity. To detect the NDH-1MS complex, the cells of WT and Δ*ndhM* were first grown at high CO_2_ and then shifted to the air condition for 24 h before isolation of the thylakoid membrane proteins. Immunoblotting analysis indicates that the NDH-1MS complex, NDH-1S complex, and free protein could be detected in the WT strain using an antibody for the key component CupA. However, in the Δ*ndhM* mutant, only the NDH-1S complex and the free CupA could be detected, whereas the NDH-1MS complex was absent in the thylakoid membranes (Fig. 3*C*). Similarly, the NDH-1MS′ complex, NDH-1S′ complex, and free protein could also be detected in the WT strain using an antibody for the key component CupB (Fig. 3*C*). In the Δ*ndhM* mutant, the accumulation of the NDH-1S′ complex and the free CupB was not affected; nevertheless, the NDH-1MS′ complex with a molecular mass of ∼550 kDa was degraded to a subcomplex of ∼400 kDa lacking the hydrophilic NDH-1 subunits in the absence of NdhM (Fig. 3*C*), implying that the entire structure of NDH-1MS and NDH-1MS′ complexes is essential for the CO_2_ uptake in *Synechocystis* 6803. These results demonstrate that the absence of NdhM causes the disassembly of the hydrophilic subcomplex of NDH-1 complexes, resulting in impairment of the NDH-1 activity.

##### Analyses of NDH-1 Subcomplex Assembly Intermediates in the Cytoplasm

**FIGURE 4.**
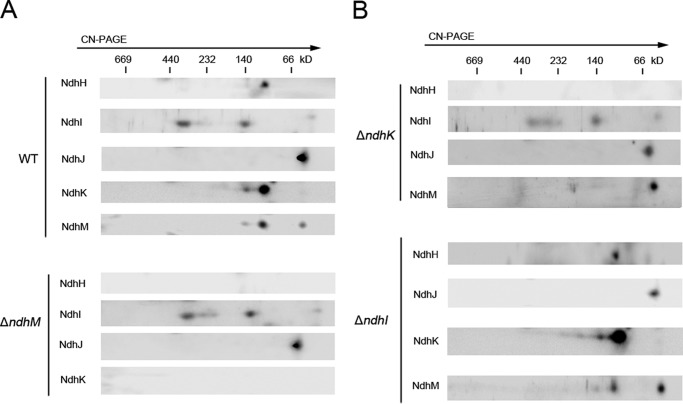
**Analysis of the cytoplasmic intermediate complexes isolated from WT, Δ*ndhM*, Δ*ndhK*, and Δ*ndhI* strains.** Cytoplasmic protein complexes isolated from WT and Δ*ndhM* strains (*A*) and Δ*ndhK* and Δ*ndhI* strains (*B*) were separated by CN-PAGE and further subjected to two-dimensional SDS-PAGE. The proteins were immunodetected with antibodies against Ndh subunits (NdhH, NdhI, NdhJ, NdhK, and NdhM). The positions of molecular mass markers are indicated.

To know whether the defect of NdhM impairs the NDH-1 assembly intermediates, we investigated the components of the NDH-1 subcomplex assembly intermediates by CN-PAGE and subsequent immunoblotting analyses for the cytoplasmic fraction. In WT, the NdhH subunit was present in a subcomplex with a molecular mass of ∼100 kDa. The NdhK and NdhM subunits were mainly present in the 100-kDa subcomplex also including NdhH, whereas they were faintly present in the ∼140-kDa subcomplex, and the free NdhM was also detected. NdhI subunit was present in two subcomplexes with molecular masses of ∼300 and ∼140 kDa which were independent of NdhM or NdhK subunit. Conversely, the NdhJ subunit was only detected as free protein (Fig. 4*A*). In the Δ*ndhM* mutant, the 100-kDa complex containing NdhH and NdhK and the 140-kDa complex containing NdhK were almost completely impaired (Fig. 4*A*), implying that NdhM is required for the formation of the NdhH and NdhK subcomplex. Only the 300- and 140-kDa subcomplexes consisting of NdhI and free NdhJ were stable in the Δ*ndhM* mutant (Fig. 4*A*).

**FIGURE 5.**
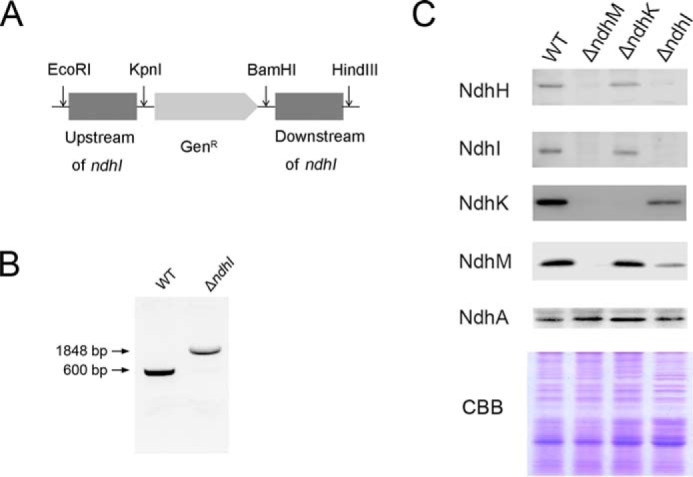
**Deletion mutation of the *ndhI* gene.** *A*, construction of plasmid to generate *ndhI* deletion mutant (Δ*ndhI*). A schematic representation of the Δ*ndhI* mutant locus is shown. A gentamicin resistance (*Gen^R^*) marker was used to replace the entire *ndhI* gene. *B*, PCR segregation analysis of the Δ*ndhI* mutant using *ndhI*-F and *ndhI*-R primers (supplemental Table 1). *CBB*, Coomassie Brilliant Blue.

To gain insights into the assembly of the cytoplasmic NDH-1 subcomplex, we further detected the hydrophilic subunits in a newly generated Δ*ndhI* mutant (Fig. 5, *A* and *B*) and in the Δ*ndhK* mutant (36). Similar to the Δ*ndhM* mutant, the 100-kDa complex containing NdhH and NdhM and the 140-kDa complex containing NdhM were undetectable in the absence of NdhK, indicating that the NdhK is important for NdhH and NdhM assembly into the NDH-1 complex in the membrane; however, free NdhM was stable in the Δ*ndhK* mutant (Fig. 4*B*). The 300- and 140-kDa subcomplexes consisting of NdhI and free NdhJ were not affected in the Δ*ndhK* mutant (Fig. 4*B*). In contrast, the NDH-1 subcomplexes containing NdhH, NdhJ, NdhK, and NdhM were not impaired in the Δ*ndhI* mutant, indicating that the assembly of these subunits was independent of NdhI in the cytoplasm (Fig. 4*B*).

##### NdhM Interacts with NdhK, NdhH, NdhI, and NdhJ Subunits

**FIGURE 6.**
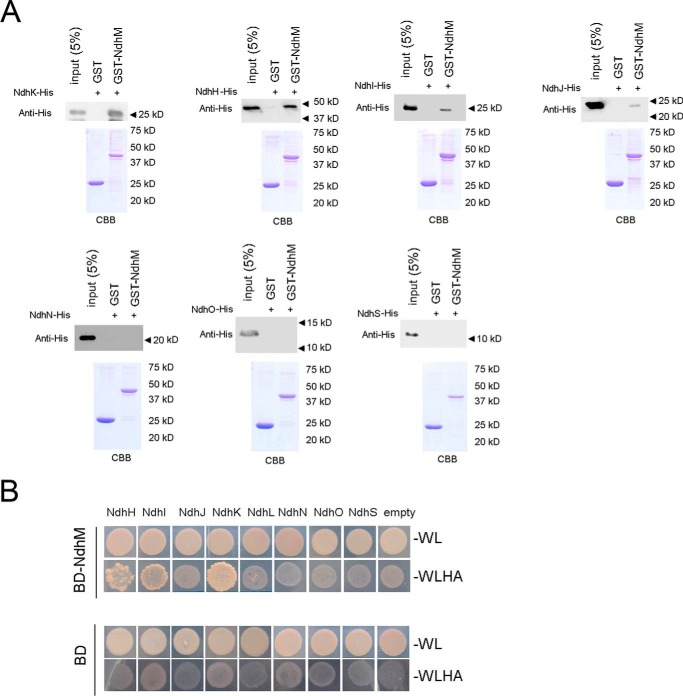
**Interaction of NdhM with NdhH, NdhI, NdhJ, and NdhK.** *A*, pulldown analysis of NdhM with the hydrophilic Ndh subunits. Recombinant His-tagged NdhH, NdhI, NdhJ, NdhK, NdhN, NdhO, and NdhS were incubated with recombinant GST or NdhM fused to GST, respectively. Proteins were bound to the GST affinity resin and then eluted with Laemmli buffer. Eluates were analyzed by SDS-PAGE and Coomassie Brilliant Blue (*CBB*) staining, and the Ndh subunits were detected by immunoblotting with anti-His antibody. *B*, yeast two-hybrid assay of the interaction of NdhM with the hydrophilic Ndh subunits. *AD*, Gal4 activation domain; *BD*, Gal4 DNA binding domain. −*WL* and −*WLHA* indicate synthetic defined medium/−Trp−Leu and synthetic defined medium/−Trp−Leu−His−Ade dropout plates, respectively. The ability to grow on −WLHA plates indicates an interaction between two proteins. Empty vectors were used as negative controls.

To investigate the role of NdhM in stabilizing cyanobacterial NDH-1 complexes, we determined the interaction of NdhM with the seven hydrophilic Ndh subunits that had been identified in the complex. We generated recombinant NdhM fused with GST. Then the recombinant NdhH-His_6_, NdhI-His_6_, NdhJ-His_6_, NdhK-His_6_, NdhN-His_6_, NdhO-His_6_, and NdhS-His_6_ fusion proteins were generated and tested for interaction with GST-NdhM, respectively. GST-NdhM was bound to a GST affinity column and used as bait. Recombinant GST was used as a negative control for unspecific binding. Proteins were eluted using Laemmli buffer and analyzed by immunoblotting (Fig. 6*A*). These *in vitro* pulldown experiments showed that NdhM strongly interacts with NdhK, NdhH, and NdhI and weakly interacts with NdhJ but does not interact with NdhN, NdhO, and NdhS subunits. Further yeast two-hybrid assay confirmed the strong protein-protein interaction between NdhM and NdhK, NdhH, and NdhI, respectively (Fig. 6*B*). We therefore conclude that NdhM interacts with NdhH, NdhI, NdhJ, and NdhK and is essential for the stability of these subunits.

## Discussion

Cyanobacterial NDH-1 complexes have multiple functions because of the diversity of the complexes based on their different subunits composition (17, 44). The NdhM subunit exclusively exists in oxygenic photosynthetic organisms (11, 33). In higher plants, knock-out of *ndhM* results in complete impairment of NDH activity and entire collapse of subcomplex A of chloroplast NDH complex (33–35). In this work, we demonstrate that NdhM is required for the NDH-1 activity and stability of the hydrophilic subunits of the complexes including NDH-1L, NDH-1M, NDH-1MS, and NDH-1MS′ in *Synechocystis* 6803. Knock-out of *ndhM* gene resulted in complete impairment of not only NDH-dependent CET around PSI (Fig. 1) but also CO_2_ uptake ability (Fig. 2*B*). Due to the loss of NDH-1L complex activity, the Δ*ndhM* mutant was unable to grow under photoheterotrophic and mixotrophic conditions (Fig. 2*A*). The mutant could not survive under air CO_2_ condition and grew a bit slower than WT under 2% CO_2_ in air, which might be caused by the disassembly of the NDH-1MS and NDH-1MS′ complexes (Fig. 3). Because all of the NDH-1 complex activities are affected in the Δ*ndhM* mutant, we suggested that NdhM is most probably a common subunit for all NDH-1 variants.

Previous study has demonstrated that the NdhL–NdhO subunits are grouped together in the central part of the membrane domain of cyanobacterial NDH-1 complex (13). However, it was suggested that NdhM is a subunit of subcomplex A of chloroplast NDH in *Arabidopsis* (34). In this work, we showed that the cyanobacterial NdhM subunit is located in the hydrophilic arm, corresponding to subcomplex A in *Arabidopsis*. In *Synechocystis* 6803, deletion of *ndhM* almost abolished the assembly of the hydrophilic subunits, such as NdhK, NdhH, NdhI, and NdhJ; however, the accumulation of the hydrophobic subunits, such as NdhA, was not affected (Fig. 3). Thus, our results indicate that, different from other hydrophilic subunits of NDH-1 complexes, NdhM serves as a core subunit of the hydrophilic subcomplex of NDH-1.

In cyanobacteria, various NDH-1 complex assembly intermediates exist in the cytoplasm including the 100-kDa subcomplex containing NdhH, NdhK, and NdhM; the 140-kDa subcomplex containing NdhK and NdhM; and the 300- and 140-kDa subcomplexes containing NdhI (Fig. 4*A*). It was shown that, in *Arabidopsis*, the formation of the NDH complex assembly intermediate consisting of NdhK was impaired in *ndhM*, whereas the formation of the intermediate including NdhH was not affected (35). In contrast, in *Synechocystis* 6803, the NdhM subunit is required for the formation of the subcomplex consisting of NdhK and NdhH; meanwhile, NdhK is essential for the formation of the subcomplex consisting of NdhM and NdhH (Fig. 4*B*). However, the accumulation of other Ndh subunits in the thylakoid membrane was not affected in the absence of NdhK (Fig. 5*C*), suggesting that in the Δ*ndhK* mutant the free NdhM and other Ndh subunits could be assembled properly in the thylakoid membrane. Besides, the NDH-1 complex assembly intermediates containing NdhH, NdhJ, NdhK, and NdhM were not affected in the Δ*ndhI* mutant (Fig. 4*B*); nevertheless, the accumulation of the NdhH, NdhK, and NdhM subunits in the thylakoid membrane was significantly impaired in the Δ*ndhI* mutant (Fig. 5*C*), indicating that the NdhI subunit is essential for the stability of other Ndh subunits in the thylakoid membrane.

**FIGURE 7.**
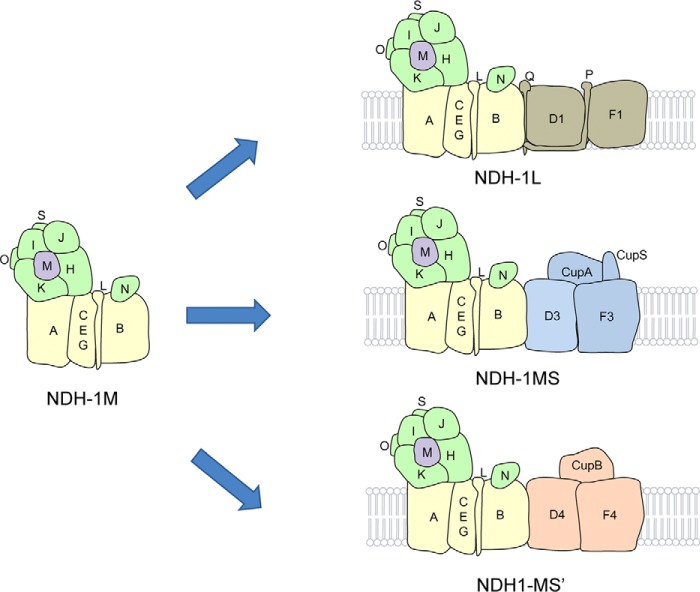
**A model schematically represents the localization of NdhM in NDH-1 complex.** The basic complex NDH-1M is combined with specific domains to assemble the functional complexes NDH-1L, NDH-1MS, and NDH-1MS′.

Based on the sequence alignment, Prommeenate *et al.* (11) suggested that NdhM is related to subunit B13 of bovine complex I. B13 is found in the hydrophilic subcomplex of complex I, designated Iλ. Recently, the architecture of complex I from *Bos taurus* heart mitochondria determined by single particle electron cryomicroscopy showed that the supernumerary B13 is adjacent to the core hydrophilic subunits 30 kDa, 49 kDa, TYKY, and PSST (45). In our study, we found that NdhM interacts strongly with NdhK, NdhH, and NdhI but does not interact with NdhL, NdhO, NdhN, or NdhS (Fig. 6). Therefore, we conclude that the NdhM subunit is localized adjacent to the hydrophilic subunits, such as NdhK, NdhH, NdhI, and NdhJ subunits, in *Synechocystis* 6803 as shown in Fig. 7. Moreover, NdhM is one of the core subunits of NDH-1M complex, which could be associated with other subcomplexes to form the NDH-1L, NDH-1MS, and NDH-1MS′ complexes. Under high CO_2_ conditions, NDH-1L is the dominant complex, whereas NDH-1MS complex is strongly induced under low CO_2_ conditions. NDH-1MS′ complex is expressed constitutively at a low level (18, 24).

In conclusion, the present study corroborated the important role of NdhM in cyanobacterial CO_2_ uptake. NdhM is located in the hydrophilic core of the NDH-1 complexes and is essential for the stability of the hydrophilic subcomplex of NDH-1MS and NDH-1MS′ as well as NDH-1L by interacting with NdhK, NdhH, and NdhI.

## Author Contributions

Z. H. carried out the main experiments and wrote the paper. M. X. helped with the transformation experiment. Y. W. carried out RT-PCR analysis. J. L. helped perform experiments. P. F. discussed the study. H. M. designed the research.

## Supplementary Material

Supplemental Data

## Footnotes

2 The abbreviations used are:

NDH-1: type I NAD(P)H dehydrogenase
CET: cyclic electron transport
PSI: photosystem I
*Synechocystis* 6803: *Synechocystis* sp. PCC 6803
NDH-1L: large size NDH-1 complex
NDH-1M: medium size NDH-1 complex
NDH-1S: small size NDH-1 complex
PSII: photosystem II
BN: blue native
CN: clear native
BisTris: 2-[bis(2-hydroxyethyl)amino]-2-(hydroxymethyl)propane-1,3-diol
*F*_0_: initial fluorescence
*F_v_*: variable fluorescence
*F_m_*: maximal fluorescence.
