# Supplementary material for: NdhM Subunit Is Required for the Stability and the Function of NAD(P)H Dehydrogenase Complexes Involved in CO2 Uptake in Synechocystis sp. Strain PCC 6803
Source: J Biol Chem. 2015 Dec 24;291(11):5902–12. doi: 10.1074/jbc.M115.698084 (PMC4786724; doi:10.1074/jbc.M115.698084)
Supplement: Supplemental Data [file supp_291_11_5902__index.html]

NdhM Subunit Is Required for the Stability and the Function of NAD(P)H Dehydrogenase Complexes Involved in CO2 Uptake in Synechocystis sp. Strain PCC 6803 — NdhM Is Required for the Function of the NDH-1 Complexes — Supplemental Data 

# NdhM Subunit Is Required for the Stability and the Function of NAD(P)H Dehydrogenase Complexes Involved in CO2 Uptake in *Synechocystis* sp. Strain PCC 6803

## Supplemental Data

**Files in this Data Supplement:**

- Supplemental Table
