# Supplementary material for: NdhM Subunit Is Required for the Stability and the Function of NAD(P)H Dehydrogenase Complexes Involved in CO2 Uptake in Synechocystis sp. Strain PCC 6803
Source: J Biol Chem. 2015 Dec 24;291(11):5902–12. doi: 10.1074/jbc.M115.698084 (PMC4786724; doi:10.1074/jbc.M115.698084)
Supplement: Supplemental Data [file supp_M115.698084_Suppltable1.pdf]

Supplemental Table 1

Primers List and Sequence

Primers used to construct the puc-Δ*ndhM* vector

Name	Sequence (5'-3')	Description
*ndh*M Up-F	CCGGAATTCCCAAAGCCGGTTTGATCAATG	Amplification of upstream region
*ndh*M Up-R	CGGGGTACCAGTAATTCTAAACAAATTTGTCCGGG	
*ndh*M Dn-F	CGCGGATCCTCAATTGACCATAACTGGAGGGAG	Amplification of downstream region
*ndh*M Dn-R	CCCAAGCTTAAGTCAGCATCATTGGTCGCTG	
Gen-F	CGGGGTACCGTTCGTAAACTGTAATGCAAGTAGCG	Amplification of gentamycine resistance gene
Gen-R	CGCGGATCCATGACGAGCGTAATGGCTGG	
ndhM-F	CCCGGACAAATTTGTTTAGAATTACT	Segregation analysis
ndhM-R	CTCCCTCCAGTTATGGTCAATTGA	

Primers used to construct the puc-Δ*ndhI* vector

Name	Sequence (5'-3')	Description
ndhI Up-F	CCGGAATTCGGGATGAAATTCGCCCTGTTC	Amplification of upstream region
ndhI Up-R	CGGGGTACCCGACGACTCATGTGGTCAAAGG	
ndhI Dn-F	CGCGGATCCGTGGCTTTGGGAAGGTTGC	Amplification of downstream region
ndhI Dn-R	CCCAAGCTTCCATGGTGCTCAACAGGGC	
ndhI-F	GGGAATTCCATATGTTTAACAACATTCTCAAACAGGTAG	Segregation analysis
ndhI-R	CGCGGATCCCTATTCTGCTTTCACCAAATCTTCG	

Primers used for RT-PCR

Name	Sequence (5'-3')	Description
ndhM-F	ATGCTTGTTAAATCCACTACCCG	*ndhM* transcript
ndhM-R	TTAGTTATCCAGCCAATATTTTCCTG	
ndhH-F	CCAGAAAAGCTAGCCGACATTG	*ndhH* transcript
ndhH-R	TTGAGGGCCTGACGAATGATC	
ndhI-F	ATGTTTAACAACATTCTCAAACAGGTAG	*ndhI* transcript

ndhI-R	CTATTCTGCTTTCACCAAATCTTCG	
ndhJ-F	TGACCACCAATGGCTTTGAG	*ndhJ* transcript
ndhJ-R	GCCAGCCTACCCAATCCTCC	
ndhK-F	ATGAGTCCCAACCCTGCTAACC	*ndhK* transcript
ndhK-R	TCAGCCACGGTTTAATTGCTC	
16 S rRNA-F	CGACTGCTAATACCCAATGTGC	*16 S rRNA* transcript
16 S rRNA-R	GTCCCTCAGTGTCAGTTTCAGC	

Primers used to construct vectors to express protein to raise NdhM antibody.

**Name**	**Sequence (5’-3’)**	**Description**
ndhM-NdeF	GGGAATTCCATATGCTTGTTAAATCCACTACCCG	NdhM antibody
ndhM-BamR	CGCGGATCCTTAGTTATCCAGCCAATATTTTCCTG	

Primers used to construct fusion protein expression vector

**Name**	**Sequence (5’-3’)**	**Description**
ndhM-EcoF	CCGGAATTCATGCTTGTTAAATCCACTACCCG	GST-tagged NdhM
ndhM-SalR	ACGCGTCGACTTAGTTATCCAGCCAATATTTTCCTG	
ndhH-F	GGGAATTCCATATGACCAAGATTGAAACCAGAACC	His_6-tagged NdhH
ndhH-R	CGCGGATCCCTAGCGGTCCACCGATCCC	
ndhI-F	GGGAATTCCATATGTTTAACAACATTCTCAAACAGGTAG	His_6-tagged NdhI
ndhI-R	CGCGGATCCCTATTCTGCTTTCACCAAATCTTCG	
ndhJ-F	GGGAATTCCATATGGTGGCTGAGGAAGTGAACTCCC	His_6-tagged NdhJ
ndhJ-R	CGCGGATCCCTAATAGGCATCCTGGAGTTCGT	
ndhK-F	GGGAATTCCATATGAGTCCCAACCCTGCTAACC	His_6-tagged NdhK
ndhK-R	CCGGAATTCTCAGCCACGGTTTAATTGCTC	
ndhN-F	GGGAATTCCATATGTTGCCATTGCCATTAATTG	His_6-tagged NdhN
ndhN-R	CGCGGATCCCTAGGCCGCCTGCAAGCA	
ndhO-F	GGGAATTCCATATGGCCGCTAAAATGAAAAAG	His_6-tagged NdhO

ndhO-R	CGCGGATCCCTAAGCCAGGGCTTCGATTTG	
ndhS-F	GGGAATTCCATATGATTTTTCCCGGTGCAAC	His_6-tagged NdhS
ndhS-R	CGCGGATCCCTAGATGGGTTTGACTGCTTCC	

Primers used to construct the yeast two-hybrid vector.

**Name**	**Sequence (5'-3')**	**Description**
ndhM-attB1	GGGGACAAGTTTGTACAAAAAAGCAGGCTCCATGCTTGTTAAATCCACTACCCG	NdhM bait
ndhM-attB2	GGGGACCACTTTGTACAAGAAAGCTGGGTTTTAGTTATCCAGCCAATATTTTCCTG	
ndhH-attB1	GGGGACAAGTTTGTACAAAAAAGCAGGCTCCATGACCAAGATTGAAACCAGAACC	NdhH prey
ndhH-attB2	GGGGACCACTTTGTACAAGAAAGCTGGGTTCTAGCGGTCCACCGATCCC	
ndhI-attB1	GGGGACAAGTTTGTACAAAAAAGCAGGCTCCATGTTTAACAACATTCTCAAACAGGTAG	NdhI prey
ndhI-attB2	GGGGACCACTTTGTACAAGAAAGCTGGGTTCTATTCTGCTTTCACCAAATCTTCG	
ndhJ-attB1	GGGGACAAGTTTGTACAAAAAAGCAGGCTCCGTGGCTGAGGAAGTGAACTCCC	NdhJ prey
ndhJ-attB2	GGGGACCACTTTGTACAAGAAAGCTGGGTTCTAATAGGCATCCTGGAGTTCGT	
ndhK-attB1	GGGGACAAGTTTGTACAAAAAAGCAGGCTCCATGAGTCCCAACCCTGCTAACC	NdhK prey
ndhK-attB2	GGGGACCACTTTGTACAAGAAAGCTGGGTTTCAGCCACGGTTTAATTGCTC	
ndhL-attB1	GGGGACAAGTTTGTACAAAAAAGCAGGCTCCATGGAAGATTTATTAGGTTTGCTACTT	NdhL prey
ndhL-attB2	GGGGACCACTTTGTACAAGAAAGCTGGGTTCTAGGCAGCCTGGCGGC	
ndhN-attB1	GGGGACAAGTTTGTACAAAAAAGCAGGCTCCATGTTGCCATTGCCATTAATTG	NdhN prey
ndhN-attB2	GGGGACCACTTTGTACAAGAAAGCTGGGTTCTAGGCCGCCTGCAAGCA	
ndhO-attB1	GGGGACAAGTTTGTACAAAAAAGCAGGCTCCATGGCCGCTAAAATGAAAAAG	NdhO prey
ndhO-attB2	GGGGACCACTTTGTACAAGAAAGCTGGGTTCTAAGCCAGGGCTTCGATTTG	
ndhS-attB1	GGGGACAAGTTTGTACAAAAAAGCAGGCTCCATGATTTTTCCCGGTGCAAC	NdhS prey
ndhS-attB2	GGGGACCACTTTGTACAAGAAAGCTGGGTTCTAGATGGGTTTGACTGCTTCC	
